# Student Health and Social Care Professionals’ Health Literacy Knowledge: An Exploratory Study

**DOI:** 10.3390/pharmacy11020040

**Published:** 2023-02-22

**Authors:** Helen Wood, Gabrielle Brand, Rhonda Clifford, Sinead Kado, Kenneth Lee, Liza Seubert

**Affiliations:** 1 School of Allied Health, The University of Western Australia, Perth 6009, Australia; 2 School of Nursing & Midwifery, Monash University, Melbourne 3800, Australia

**Keywords:** health literacy, health care professionals, social care professionals, health education

## Abstract

Health literacy is essential for shared decision-making and improved health outcomes, and patients with inadequate health literacy often need additional support from health and social care professionals. Despite global calls for developing tertiary-level health literacy education, the extent of this in Australian health and social care professional degrees is unknown. This research explored students’ health literacy knowledge across five health and social care professional disciplines. A web-based questionnaire was disseminated to student health and social care professionals enrolled in one of two Australian universities. Questions explored students’ factual and conceptual health literacy knowledge, and responses were inductively themed and reported descriptively. Of the 90 students who participated, the depth of health literacy knowledge was low. Students frequently identified understanding as components of health literacy; however, most students did not identify health information access, appraisal and use. Additionally, students’ knowledge of helping patients with inadequate health literacy was limited. Adjusting patient education to their health literacy level and evaluating patient understanding was poorly understood. Without a solid understanding of fundamental health literacy principles, newly-graduated health and social care professionals will be poorly equipped to facilitate patients’ health literacy-related challenges in the community. Further exploration of health literacy education is urgently recommended to identify areas for improvement.

## 1. Introduction

Health literacy is a broad concept that describes an individual’s capacity to access, understand, appraise and use health information in a beneficial way [1]. It plays a critical role in achieving positive health outcomes. For example, a person with adequate health literacy is better equipped to take actions that support their own health, such as navigating health and social care systems, understanding health messages and engaging in shared decision-making [1,2,3]. The reverse is also true; a person with inadequate health literacy may struggle to communicate effectively with a health or social care professional, understand health communication or correctly adhere to treatment [1,4,5]. This has been associated with negative health outcomes, such as longer recovery times, development of chronic health conditions or earlier death [4,5,6,7].

While individual factors, such as age or cultural background, influence health literacy [6,8,9,10], the role of health and social care professionals cannot be understated. As first (and often, only) point of patient contact, their professional responsibility extends to ensuring equitable and accessible healthcare for all patients, across the spectrum of individual health literacy capabilities.

There is a plethora of evidence, arising primarily from the United States, demonstrating the positive impact that health or social care professionals can have on a patient’s ability to manage their health; initiatives such as ‘Ask Me 3’, ‘Universal Precautions Approach’ or teach-back methods have concentrated on health literacy strategies which help health and social care professionals support patient engagement and understanding [11,12,13,14,15,16]. By implementing specific health literacy-related initiatives, health and social care professionals can improve patients’ treatment adherence [14,17], health-related knowledge [14,16,18,19], patient satisfaction [15,19,20], and health outcomes [16,21,22].

In 2011, an international panel of health literacy advocates published the Calgary Charter on Health Literacy, which proposed the need for developed and evaluated health literacy curricula for health profession students [23]. Similarly, Australia’s National Statement on Health Literacy identified that health literacy education must be integrated into health professionals’ curricula as part of a national action plan—a position recently endorsed by the Australian Medical Association and the World Health Organization [1,24,25]. However, the extent of health literacy education in Australian health and social care professional degrees is largely still unknown. To date, one Australian study evaluated an implemented health literacy curriculum for student pharmacists, using a novel scaffolded education activity where students created and delivered an education session to small patient groups [26]. The success of the education activity was measured using teacher, student and patient evaluations. While promising, this highlights the lack of Australian research that evaluates the health literacy curricula in a range of health and social care professional degrees.

When proposing a health literacy training framework, Saunders et al. identified health literacy knowledge as the first of a series of learning outcomes essential to a curriculum, aiming to improve student attitude, knowledge and skills, social health care quality, and patient capacity and satisfaction [27]. This aligns with the position of knowledge as the first step in Bloom’s revised taxonomy, a hierarchical model used to classify components of educational learning [28]. The model proposes that knowledge is the foundation of all learning which students must master in order to attain competence in more complex components [28].

The purpose of this exploratory research was to explore university students’ health literacy knowledge across five health and social care professional disciplines. This will provide a unique insight into students’ interpretation of the current curricula, which is the first step towards the identification of key strategies to help better align health literacy curricula with the National Statement on Health Literacy.

## 2. Materials and Methods

### 2.1. Overview

A cross-sectional, web-based questionnaire was chosen for data collection, as it allowed participation from students who may wish to retain anonymity or avoid scrutiny from the researchers [29]. It was disseminated to final-year student health and social care professionals, to gain insight into their understanding of the term ‘health literacy’ and knowledge of its importance to both their patients and themselves as future health and social care professionals. The Human Research Ethics Office at The University of Western Australia granted approval for the study (RA/4/20/5960). All data collected were managed and stored securely as per ethics requirements.

### 2.2. Questionnaire Development

The questionnaire was created by health professional academics with expertise in health literacy (HW, GB, KL), giving consideration to previously-identified health literacy competencies for health professionals [27,30,31]. To determine students’ level of health literacy knowledge, questions were mapped to the first two dimensions of Bloom’s revised taxonomy of educational learning objectives: factual knowledge and conceptual knowledge [28]. Factual knowledge relates to the basic definitions of a concept which familiarize students with the topic. Conceptual knowledge extends further to the principles and categories which underpin that topic [28]. The questionnaire, provided in Supplementary material SA, was created in Qualtrics (Qualtrics, Provo, UT, USA), an internet-based questionnaire platform, and comprised two sections:

1. Demographics and their understanding of the term ‘health literacy’ encompassing the first dimension, or ‘factual knowledge’. It asked for words or phrases that students think of when they hear the term ‘health literacy’.
2. Conceptual health literacy knowledge encompassing the second dimension, or ‘conceptual knowledge’. This section asked for three signs of inadequate patient health literacy, three potential consequences of inadequate patient health literacy, and three actions that health and social care professionals can take to help a patient with inadequate health literacy.

Additional space was provided at the conclusion of the questionnaire for students to write any comments on the health literacy education embedded within their curriculum. For all questions, responses were collected in open text boxes. The consent statement, with a single option to select ‘yes’, required an affirmative response to continue. All other questions prompted for a response, but students were able to leave questions unanswered. The first page sought the students’ understanding of the term ‘health literacy’; each subsequent page provided the following definition of health literacy to ensure all conceptual knowledge questions were answered with the same definition in mind: ‘Health literacy is the degree to which individuals have the capacity to obtain, process and understand basic health information and services needed to make appropriate health decisions’ [32]. The ‘back’ button was disabled on the questionnaire, so students could not amend their original understanding of health literacy based on the provided definition. Participants were prevented from submitting multiple responses by utilizing a function of the survey tool which blocks subsequent access attempts.

The questionnaire was piloted with five student health professionals to ensure the questions were appropriate and easily understood, and that online formatting worked as intended on different devices. No changes were recommended during piloting, and data generated from piloting were excluded from analysis.

### 2.3. Participants and Recruitment

The target population included students enrolled in the following health disciplines from one of two Western Australian universities: Dental Medicine, Nursing, Pharmacy, Podiatric Medicine, and Social Work and Social Policy. Prior to recruitment, a member of the research team (HW) contacted academic staff from each health or social care discipline to identify potential student cohorts who had completed all health literacy-related content. Students were eligible to participate if they consented and were:

- Aged 18 years or older;
- Currently enrolled in one of the aforementioned health or social care disciplines;
- Able to read and write in English;
- Advanced enough through their degree that they had completed all health literacy-related content at the time of participation.

Age, enrolment status and English language capabilities were assured due to recruiting students in the final year of their degree in an English-speaking university. Completion of health literacy-related content was verified by targeted recruitment to students in their final year at a time-point recommended by the relevant academic staff; a question in the demographics section relating to their progression through the degree was used to double-check eligibility. Recruitment was conducted through broadcasted announcements on Learning Management System, which could be viewed through the software or via email. A reminder announcement was disseminated one week prior to the survey closing.

Potential stress or perceived obligation to participate could have existed due to an unequal relationship between some research members and participants, as some researchers were known to potential participants; this was mitigated using an anonymous, web-based questionnaire and voluntary participation. Additionally, research members did not directly approach any student to encourage or request participation. Students had the opportunity to win an AUD25 e-gift card following participation, but survey responses were not linked to contact information.

### 2.4. Sample Size

As this was an exploratory study where a wide range of answers was anticipated, we did not aim for data saturation to determine sample size. Rather, we aimed for a mid-range sample size of between 60 and 99 participants [33].

### 2.5. Analysis and Reporting

Anonymous data collected via Qualtrics were automatically generated into a report after data collection was completed. Free-text responses in the questionnaire were inductively themed following a manifest content analysis process. Two researchers (HW and SK, a pharmacy educator and health professions educator, respectively) independently followed a modified version of the process, described by Kleinheksel et al. [34]; after data immersion, a codebook was developed by HW for each of the four core knowledge questions. The codebook was developed iteratively and each response was re-coded when an amendment was made to the codebook.

Each codebook contained a:

- Category: each response was linked to at least one relevant category. The category ‘Vague’ was used when a response was ambiguous and the research team could not definitively derive the intent with context (e.g., ‘Impacts other areas’ as a potential consequence of inadequate health literacy could have been interpreted in multiple ways, with no context to derive intended meaning). The category ‘Other’ was used when a response did not fit within an existing category and was not reported frequently enough to warrant its own category (e.g., ‘Stress in health services’ as a sign of inadequate patient health literacy). One-word answers were categorized verbatim (e.g., the response ‘Understanding’ was categorized as ‘understanding’); when students provided more than one word the response was linked to a separate, more descriptive category (e.g., the response ‘Understanding colds and flu’ was categorized as ‘understanding health generally’).
- Code: within each category, similar responses were grouped together into codes. A single response could be attached to multiple codes (e.g., ‘Feel unable to navigate health system and end up avoiding health appointments’ was linked to ‘Not knowing where to go’ and ‘Low engagement with health professional’). The code ‘Other’ was used when a response fit within a category, but was not mentioned frequently enough to warrant its own code (e.g., ‘Impatience’ as a code within ‘Patient demeanor’).
- Description: a description of each category or code to ensure consistent theming between researchers.
- Key example: an example of a direct quote to enhance the description of the category or code.

For the question, ‘List three actions that you as a health or social care professional can take to assist a patient with inadequate health literacy’, some codes had so many responses that themes emerged within a code; when this occurred, sub-codes were created.

After coding a random 20% of responses using each draft codebook, HW and SK met to compare analyses and discuss inconsistencies. Three codebooks contained no inconsistences and one codebook contained two inconsistencies. The two researchers were able to reach agreement on the appropriate coding without need for moderation; the description of the code was updated to provide clarity. Previously coded data were double-checked by both researchers to ensure the updated description did not introduce further inconsistencies. After coding the full data set, HW and SK met again to compare analyses. There was disagreement with four responses, where one researcher applied a known code and the other used the category “vague”. To remove potential that researcher bias, experience or knowledge had influenced the data analysis of the four responses, the decision was made to defer to the judgment of the researcher who felt it was too vague to accurately code.

## 3. Results

### 3.1. Demographics

A total of 137 responses were recorded; of those, 90 were submitted as complete and eligible for inclusion. Participating students were all in their final year of study, were more likely to be female (*n* = 61; 68%), 23 years (interquartile range 2) and enrolled in a pharmacy program (*n* = 31; 34%) (Table 1).

### 3.2. Understanding of the Term ‘Health Literacy’

**Figure 1:**
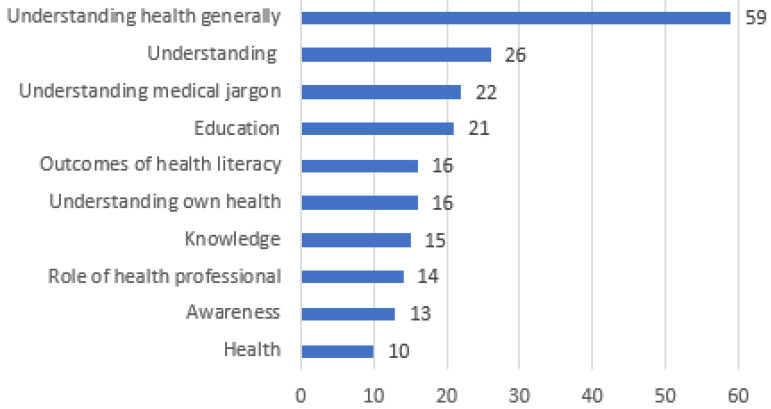
Ten most frequently reported words or phrases relating to students’ understanding of health literacy.

A total of 352 health literacy-related words or phrases were assigned to a category; the ten most frequently mentioned categories are shown in Figure 1 (the complete list is available in Supplementary material SB). Each student provided an average of four words or phrases, demonstrating they believe that health literacy relates to understanding health generally (*n* = 59; 17%), understanding (*n* = 26; 7%), understanding medical jargon (*n* = 22; 6%), and education (*n* = 21; 6%).

### 3.3. Three Signs Which Suggest Inadequate Patient Health Literacy

**Figure 2:**
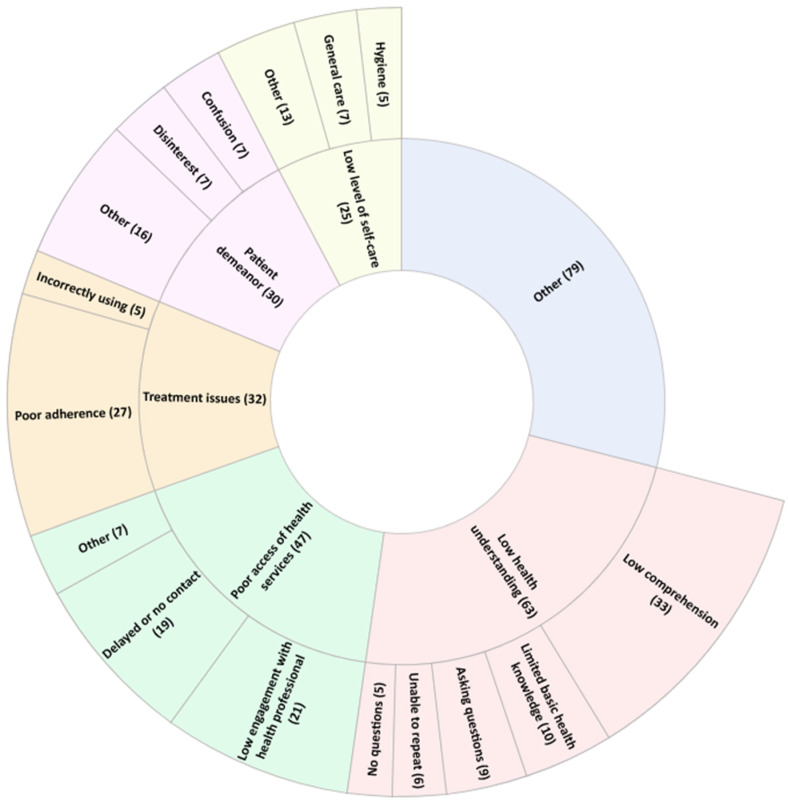
Student-described patient signs which suggest inadequate health literacy. The five most frequently reported categories, and ‘Other’, are displayed. The inner wheel lists the category; the outer wheel lists the codes within that category. The number of times a response was assigned to a given category or code is reported in brackets. The category ‘Other’ denotes all responses which did not fit within the top five categories. The code ‘Other’ denotes all codes which contain less than 2% (*n* = 5) of total responses for the question.

When asked to name three signs which suggest inadequate patient health literacy, students provided 276 responses across 11 categories. The five most frequently provided categories are published in Figure 2 (the complete list is available in Supplementary material SC). The three most mentioned coded signs that could suggest inadequate patient health literacy, as described by students, were low comprehension (*n* = 33; 12%), poor adherence (*n* = 27; 10%); and low engagement with health professionals (*n* = 21; 8%).

### 3.4. Three Potential Consequences of Inadequate Patient Health Literacy

**Figure 3:**
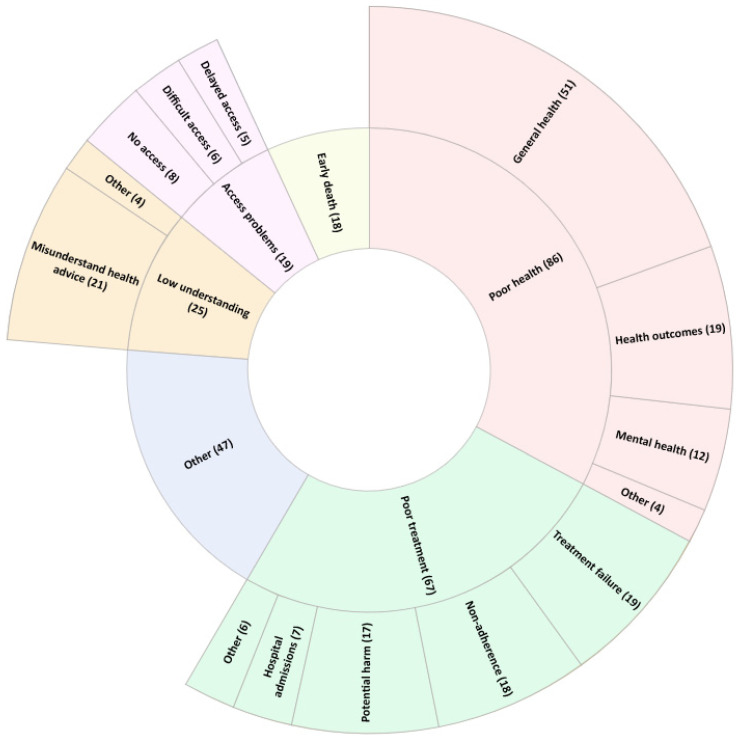
Student-described potential consequences of inadequate patient health literacy. The five most frequently reported categories, and ‘Other’, are displayed. The inner wheel lists the category; the outer wheel lists the codes within that category. The number of times a response was assigned to a given category or code is reported in brackets. The category ‘Other’ denotes all responses which did not fit within the top five categories. The code ‘Other’ denotes all codes which contain less than 2% (*n* = 4) of total responses for the question.

Students were asked to describe three potential consequences of inadequate patient health literacy, resulting in 262 responses, across 11 categories. The five most frequently provided categories are shown in Figure 3 (the complete list is available in Supplementary material SD). Students described poor general health (*n* = 51; 19%), misunderstanding health advice (*n* = 21; 8%) and treatment failure (*n* = 19; 7%) to be consequences of inadequate patient health literacy.

### 3.5. Three Actions That Health and Social Care Professionals Can Take to Help a Patient with Inadequate Health Literacy

**Figure 4:**
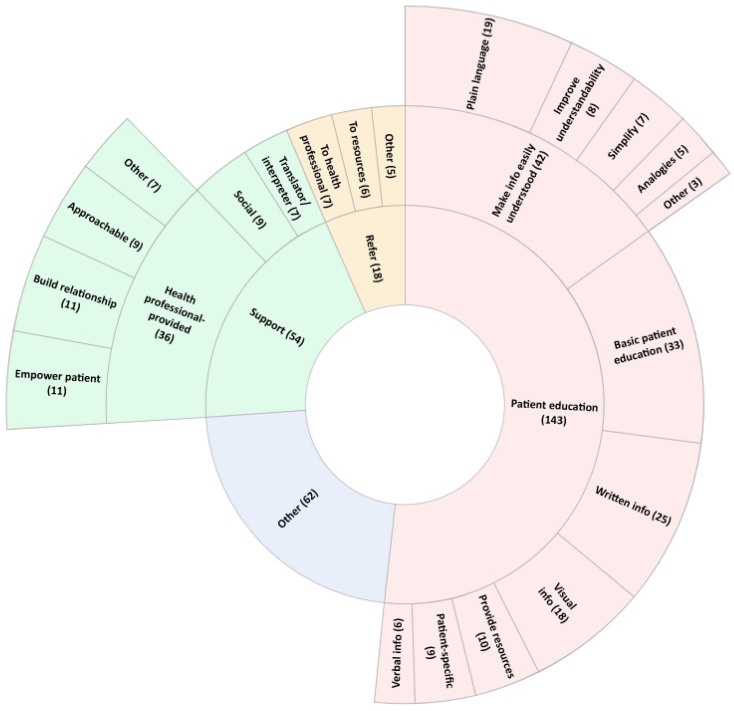
Student-described actions that a health or social care professional can take to help a patient with inadequate health literacy. The three most frequently reported categories, and ‘Other’, are displayed. The inner wheel lists the category; the middle wheel lists the codes within that category and the outer wheel lists sub-codes from the three most frequently reported codes, where applicable. The number of times a response was assigned to a given category, code or sub-code is reported in brackets. The category ‘Other’ denotes all responses which did not fit within the top five categories. The code and sub-code ‘Other’ denote all codes and sub-codes which contain less than 2% (*n* = 5) of total responses for the question.

When asked to name three actions that a health and social care professional can take to help a patient with inadequate health literacy, students provided 277 responses across ten categories. The three most frequently provided categories are published in Figure 4 (the complete list is available in Supplementary material SE). Students identified the need to make health information easily understood (*n* = 42; 15%), have health professional-provided support (*n* = 36; 13%) and provide basic patient education (*n* = 33; 12%). Of the 49 suggestions to provide written, visual or verbal education material, nine (18%) mentioned ensuring the provided material was understandable to the patient.

### 3.6. Additional Comments

Thirty students wrote additional comments about health literacy within their curriculum (33%). Of these, two students (7%) were satisfied with the current level of health literacy education (‘I think the classes on health literacy in my current degree have been very informative and helpful for us students to have a real understanding of it’). Twenty-eight comments (93%) saw students identify gaps in their own understanding (‘now that I reflect on it I feel like we have under-prepared for this’). Fifteen comments (50%) identified specific areas that students would appreciate more learning on (‘Most of our simulated patients have moderate health literacy, it would be good to have a wider range of health literacy levels. This will allow us to practice adapting our information gathering and counselling methods’). Four comments (13%) linked students’ desire for increased health literacy content to a real-world context (‘This is a very important part of being a health practitioner especially in the context of multicultural Australia’).

## 4. Discussion

To our knowledge, this is the first study worldwide that has explored student health and social care professionals’ factual and conceptual knowledge of health literacy, starting with their self-described definition of the term. It provides significant and interesting insights into students’ health literacy knowledge following the completion of all relevant curriculum components. The results suggest students had limited understanding of knowledge after completing the health literacy curriculum.

As Bloom’s taxonomy identifies, the first fundamental component of a set of learning objectives is knowledge of topic-related definitions [28]. The majority of students did not describe three of the four key components of health literacy—how patients are able to access, appraise and use health information [1]. The fourth component—understanding—did not appear to be well understood, with many students believing it related to understanding general health conditions rather than understanding relevant health information. As understanding of the term ‘health literacy’ was poorly understood in our study cohort, it suggests that health literacy needs to be more explicitly taught in curricula. If students do not understand the definition of health literacy, they do not have the foundational knowledge and understanding necessary for developing their health literacy capabilities as health and social care professionals. Furthermore, we know that health and social care professionals are in a unique position to facilitate patients’ ability to access, understand, appraise and use health information [1,35,36,37]. If students are unaware that the scope of health literacy extends as broadly as it does, as practicing health and social care professionals, they will inadvertently miss many opportunities to have a positive impact on facilitating the full spectrum of patients’ health literacy that impact their long-term health outcomes.

Once prompted with a definition of health literacy, students demonstrated some understanding of signs and behaviors of patients with, and potential consequences of, inadequate health literacy. Particularly, many students understood that patients with inadequate health literacy could display low comprehension, reduced engagement with a health professional and poor adherence, all of which are well-known signs of inadequate health literacy [1,4,5]. Similarly, students were aware of commonly-reported consequences of inadequate patient health literacy, such as treatment failure, patient misunderstanding and poorer health outcomes [4,5,6]. However, it was evident that they were unable to describe effective strategies to assist their identification of, and help patients with, inadequate health literacy. Students continued to focus their responses on the ‘understanding’ aspect of health literacy and were more likely to describe a ‘one-size-fits-all’ approach to patient education, where they would supply health information without considering other relevant patient factors, such as their ability to understand the message. Of the responses which addressed patient understanding, it was often suggested to measure understanding through closed questioning or creating a perception that they were open to answering patients’ questions if asked. This strategy is likely to be less effective as patients tend to avoid asking questions or saying they do not understand [1,38,39], a phenomenon more likely to be observed in groups who are at higher risk of having inadequate health literacy (such as older adults) [39]. This gap in students’ knowledge could be addressed by teaching core health literacy principles, such as the teach-back method, an effective way to gauge patient understanding, or the Ask Me 3 initiative, to empower patients to ask important questions designed to further their understanding [11,12,13,14].

From the additional comments, it is apparent that students recognize gaps in health literacy knowledge and, by extension, their own capabilities. A third of students revealed that they would welcome additional opportunities to learn not only the factual and conceptual knowledge around health literacy, but higher dimensions of Bloom’s taxonomy where they learn to apply new knowledge and skills with simulated and/or real patients.

When considering the ‘knowledge’ component of published health literacy competencies for health professionals [27,30,31], the student health and social care professional participating in this exploratory research may not yet meet the requisite health literacy knowledge recommended by these publications. If gaps in knowledge do indeed exist, this could have implications for students’ ability to develop their health literacy capabilities in more complex learning components.

### 4.1. Strengths and Limitations

The use of an anonymous, web-based questionnaire helped create an environment where students felt safe to provide authentic responses in their own words, rather than choosing or guessing from a list of pre-defined statements. The use of two independent researchers for data analysis provided confirmability to the results.

As this was an exploratory study, data were collected from two universities in the same large metropolitan area of Western Australia. There was also unequal representation from each discipline within the study cohort. These two factors limit the transferability of the results; however, the inclusion of five separate health and social care disciplines lessens this impact. We were also unable to access the number of prospective participants enrolled in each degree, so we cannot provide insight into the response rate to the survey.

We were unable to access details of the complete health literacy curriculum for all disciplines, so participant eligibility was partly guided by course directors who knew the health literacy curriculum throughout the degree. As we did not have access to specific education activities, we are unable to comment on the completeness of the current health literacy curricula. Nevertheless, whether gaps exist in the curriculum content or in students’ interpretation—or a mix of both—this study has highlighted that further investigation in this area is necessary.

Finally, questionnaire responses were in the form of a word, phrase or short statement. The brevity of responses meant that data was not as rich as is typical for qualitative research, and contextual information or participant thoughts around the responses were missing. There is potential that participants provided a short-form response which did not reflect their deeper understanding, and future research should utilize a different methodology to overcome this potential limitation.

### 4.2. Future Research

This research is the first step towards a deeper understanding of university students’ health literacy knowledge. Further research is recommended to gain insight into students’ health literacy capabilities within more advanced dimensions of Blooms’ revised taxonomy. Additionally, findings from this research can be used to inform both quantitative research, using a larger participant cohort from other universities and disciplines, and further qualitative research, exploring students’ reasoning behind each response. Insight from the recommended research will identify opportunities to improve upon the current health literacy-related education.

## 5. Conclusions

Health and social care professionals play an important role in supporting patients’ health literacy development, but their usefulness is hindered without comprehensive health literacy capabilities. Major gaps were uncovered when exploring student health and social care professionals’ factual and conceptual health literacy knowledge; generally, students were unable to articulate key components of the definition of health literacy and were largely unable to suggest appropriate recommendations to assist patients with inadequate health literacy. This highlights an important extension to the National Statement on Health Literacy—although including health literacy education in health and social care professions curricula is essential, so too is a robust evaluation of the education’s effectiveness. Creating evidence-based and evaluated health literacy education for university will solidly pave the way for future health and social care professional graduates to have the increased capability to achieve better health outcomes for patients across the health literacy spectrum.

## Figures and Tables

**Table 1:** Student demographics; IQR = Interquartile range.

Age	Median 23 Years (IQR 2)	
Gender *n* (%)	Female	61 (68%)
	Male	27 (30%)
	Undisclosed	2 (2%)
Health discipline *n* (%)	Pharmacy	31 (34%)
	Podiatric Medicine	26 (29%)
	Social Work and Social Policy	18 (20%)
	Nursing	8 (9%)
	Dental Medicine	7 (8%)

## Data Availability

Not applicable.

## Supplementary Materials

The following are available online at https://www.mdpi.com/article/10.3390/pharmacy11020040/s1, Supplementary material SA: Survey questions, Supplementary material SB: Ten most frequently reported words or phrases relating to students’ understanding of health literacy, Supplementary material SC: Three student-reported signs which suggest inadequate patient health literacy, Supplementary material SD: Three student-reported potential consequences of inadequate patient health literacy, Supplementary material SE: Three student-reported actions that health and social care professionals can take to help a patient with inadequate health literacy.
