# Supplementary material for: Student Health and Social Care Professionals’ Health Literacy Knowledge: An Exploratory Study
Source: Pharmacy (Basel). 2023 Feb 22;11(2):40. doi: 10.3390/pharmacy11020040 (PMC10037638; doi:10.3390/pharmacy11020040)
Supplement: Supplementary file 1 [file pharmacy-11-00040-s001.zip › pharmacy-2176160-supplementary.pdf]

Supplementary material A. Survey questions

1. How old are you?
2. Which gender do you identify with?
    a. Male
    b. Female
    c. Other
    d. Prefer not to say
3. Which degree program and year are you currently enrolled in?
4. Please list the words and/or phrases that come to mind when you hear the term "health literacy"

[new page]

For the rest of this survey, please use the following definition of health literacy when considering your responses: **"Health literacy is the degree to which individuals have the capacity to obtain, process and understand basic health information and services needed to make appropriate health decisions".**

5. What are three signs or behaviours in patients which could indicate inadequate health literacy?
    a.
    b.
    c.

[new page]

For the rest of this survey, please use the following definition of health literacy when considering your responses: **"Health literacy is the degree to which individuals have the capacity to obtain, process and understand basic health information and services needed to make appropriate health decisions".**

6. What are three consequences that patients might experience because of their inadequate health literacy?
    a.
    b.
    c.

[new page]

For the rest of this survey, please use the following definition of health literacy when considering your responses: **"Health literacy is the degree to which individuals have the capacity to obtain, process and understand basic health information and services needed to make appropriate health decisions".**

7. What are three things that you, as a health professional, can do to help someone with inadequate health literacy?

a.
b.
c.

[new page]

8. Is there anything you would like to add about the health literacy curriculum in your current degree program?

Supplementary material B: Ten most frequently reported words or phrases relating to students' understanding of health literacy

| CATEGORY | COUNT | EXAMPLE RESPONSE |
|---|---|---|
| **Understanding health generally** | 59 | An individual's knowledge of common health conditions |
| **Understanding** | 26 | Understanding |
| **Understanding medical jargon** | 22 | How well you understand medical jargon |
| **Education** | 21 | Education |
| **Outcomes of health literacy** | 16 | Decreased health literacy negatively impacts health outcomes |
| **Understanding own health** | 16 | Knowledge of one's own health condition |
| **Knowledge** | 15 | Knowledge |
| **Role of health professional** | 14 | Clinical expertise |
| **Awareness** | 13 | Awareness of health issues |
| **Health** | 10 | Health |
| **Access** | 8 | How people are able to access health information |
| **Appraisal** | 7 | Being able to identify fact from myth |
| **Patients** | 6 | Patients |
| **Using health information** | 6 | Follow advice accordingly to aid in their health/recovery journey |
| **Compliance** | 5 | Compliance |
| **Comprehension** | 5 | Comprehension |
| **Educated about health** | 5 | Education around health issues |
| **Understanding community health** | 5 | Knowing what is the situation with Covid |
| **Communication** | 4 | Communication |
| **Functional literacy** | 4 | Reading |
| **Vague** | 25 | [This code was used when the intention of the response was ambiguous] Conditions |
| **Other** | 60 | [This code was used when less than four responses (1% of total responses) were linked to a named code] Papers written on health research |

Supplementary material C: Three student-reported signs which suggest inadequate patient health literacy

| CATEGORY | CODE | EXAMPLE RESPONSE |
|---|---|---|
| **Low health understanding (63)** | Low comprehension (33) | Poor knowledge of medication they take |
| | Limited basic health knowledge (10) | Not understanding basic health information |
| | Asking questions (9) | They ask a lot of questions about their condition |
| | Unable to repeat (6) | Appear confused when you ask them to repeat instructions back |
| | No questions (5) | Simply saying 'yes' or agreeing to everything that is said without asking questions or clarifying information |
| **Poor access of health services (47)** | Low engagement with health professional (21) | An unwillingness to listen to the information provided by the pharmacist |
| | Delayed or no contact (19) | Unwilling to see doctor or go to hospital when referred |
| | Not knowing where to go (3) | Lack of knowledge of available health/support services |
| | Incorrect registration (3) | Incomplete or inaccuracy in past and current medical history |
| | Other (1) | Repeated admissions to hospital |
| **Treatment issues (32)** | Poor adherence (27) | Poor compliance with health interventions |
| | Incorrectly using (5) | Not taking their medication properly |
| **Patient demeanour (30)** | Disinterest (7) | Showing a lack of interest in their own healthcare |
| | Confusion (7) | Looking confused when being explained |
| | Frustration (4) | Getting frustrated or upset when trying to understand health or medication related concepts |
| | Other (12) | Impatience |
| **Low level of self-care (25)** | General care (7) | Engaging them in activities that could harm them and their condition |
| | Hygiene (5) | Poor personal hygiene |
| | Diet (4) | Unhealthy eating behaviours – leading to diabetes/obesity |
| | Other (9) | Extreme BMI (low or high) |
| **Patient background (24)** | Languages understood (8) | Doesn't speak English fluently |
| | Completed education (5) | Minimal education |
| | Socioeconomic status (5) | Low socioeconomic area |
| | Functional literacy (4) | Reading and writing difficulties |
| | Other (2) | Being new to Australia and not knowing the system |
| **Poor health (12)** | General health (5) | Poor health |
| | Cognitive health (3) | Cognitive decline |
| | Other (4) | Mental health disorder |
| **Non-traditional health beliefs (11)** | Appraising information (8) | When they do not scrutinize their own mislead beliefs |
| | Using information (3) | Medicating themselves with non scientific research |
| **No shared decision-making (5)** | | Refusing all treatment options |
| **Vague (22)** | | Ignorant |
| **Other (5)** | | Stress in health service |

Supplementary material D: Three student-reported potential consequences of inadequate patient health literacy

| CATEGORY | CODE | EXAMPLE RESPONSE |
|---|---|---|
| **Poor health (86)** | General health (51) | Prone to more infections/diseases |
| | Health outcomes (19) | Poorer health outcomes for themselves |
| | Mental health (12) | Experience anxiety or depression when dealing with health issues |
| | Emotional health (3) | Emotional harm may be brought upon patients |
| | Other (1) | It may affect their family's health (such as the antivaxxer movement) |
| **Poor treatment (67)** | Treatment failure (19) | They may not receive any therapeutic benefit from treatments |
| | Non-adherence (18) | Not adhering to their medications, get further complications |
| | Potential harm (17) | Inappropriate use of medication resulting in harm |
| | Hospital admissions (7) | Numerous hospital admissions |
| | No follow-up (3) | Not coming in for reviews |
| | Other (3) | Consistently seeking health care |
| **Low understanding (25)** | Misunderstand health advice (21) | Misunderstanding of treatment directives which could negatively impact their health |
| | Spread health misinformation (3) | Promote incorrect health information to family and friends |
| | Other (1) | Believing misinformation |
| **Access problems (19)** | No access (8) | Avoidance of health care |
| | Difficult access (6) | Difficulty in accessing services |
| | Delayed access (5) | Cannot receive timely health support |
| **Early death (18)** | | Decreased life expectancy |
| **Health literacy environment problems (11)** | | Poor rapport with health professionals |
| **Reduced quality of life (7)** | | Lower quality of life |
| **Financial consequences (5)** | | Large unnecessary financial costs |
| **Poor appraisal skills (5)** | Unable to make informed health decisions (5) | Unable to make informed decisions regarding their own health care |
| **Social consequences (3)** | | Trouble with relationships between friends, family and partners |
| **Vague (14)** | | Impacts other areas |
| **Other (2)** | | Incorrect diagnosis |

Supplementary material E: Three student-reported actions that health and social care professionals can take to help a patient with inadequate health literacy.

| CATEGORY | CODE | SUB-CODE | EXAMPLE RESPONSE |
|---|---|---|---|
| **Patient education (143)** | Make info easily understood (42) | Plain language (19) | De-technicalise terms and treatments |
| | | Improve understandability (8) | Provide information at a level that fits with them |
| | | Simplify (7) | Explain things in a simple manner |
| | | Analogies (5) | Relating to things they are familiar with |
| | | Speak slowly (3) | Speak slowly so they understand |
| | Basic patient education (33) | | Provide them with information |
| | Written (25) | Provide (15) | Give written information that they can refer back to |
| | | Create (5) | Write down notes for them to take home |
| | | Ensure understandability (5) | Provide easy to understand infographics |
| | Visual (18) | Provide (12) | Provide visual material eg pamphlets |
| | | Ensure understandability (4) | Provide information in a visual manner for the client to understand |
| | | Create (2) | Draw diagrams |
| | Provide resources (10) | | Provide the right resources to help them learn |
| | Verbal (6) | Provide (5) | Provide verbal counselling/information |
| | | Ensure understandability (1) | Use words that can help a patient to understand the information |
| | Patient-specific (9) | | Educate them on their condition, treatments and potential benefits |

| | | | |
|---|---|---|---|
| **Support (54)** | Health professional-provided (36) | Build relationship (11) | Build rapport with patients |
| | | Empower patient (11) | Build capacity for help seeking |
| | | Approachable (9) | Do not make them feel bad about this/judge them |
| | | More contact (4) | Follow up with these people, to make sure they are still understanding what to do |
| | | Administrative (3) | Assist in completing forms |
| | Social (9) | Educate (5) | Provide education and support to their significant others or family members |
| | | Enlist (3) | Seeing if there are family and friends who can support them |
| | | Explore (1) | Examine the person's social system and connections |
| | Translator/interpreter (7) | | Find a way to translate key information |
| **Refer (18)** | To resources (6) | | Guide them to resources for education |
| | To health professional (7) | | Refer to another professional e.g. diabetes educator |
| | General (5) | | Provide referral |
| **Evaluate understanding (12)** | Ask questions (8) | | Ask them if they know the consequences of not being compliant |
| | Ask patient to repeat (4) | | Get the patient to recite back information that was just explained to them |
| **Understand patient perspective (9)** | | | Envision yourself in the position of a patient |
| **Give time (7)** | | | Take the time to explain their diagnosis |
| | Directly (6) | | Ask if they need help |

| | | | |
|---|---|---|---|
| **Allow questions (10)** | Indirectly (4) | | Create an environment where people can ask questions |
| **Improve their health literacy (3)** | | | Educate about the importance of health literacy |
| **Vague (12)** | | | Assist |
| **Other (9)** | | | Advocate for improving health literacy within the community |
